# One-Pot Synthesis of Pd@Pt Core-Shell Icosahedron for Efficient Oxygen Reduction

**DOI:** 10.3390/ma18061279

**Published:** 2025-03-13

**Authors:** Zisheng Tang, Dafu Zhao, Xiaoqian Wang, Yanhui Jiao, Manrui Liu, Chengqi Liu, Qi Zhang, Shujing Ren, Yong Liu

**Affiliations:** State Key Laboratory of Advanced Technology for Materials Synthesis and Processing, Wuhan University of Technology, Wuhan 430070, China; tangzs3076@163.com (Z.T.); dafu484@whut.edu.cn (D.Z.); 303568@whut.edu.cn (X.W.); yanhui.jiao@outlook.com (Y.J.); liumanr14@163.com (M.L.); liuchengqi42@163.com (C.L.); zq13307239180@163.com (Q.Z.); rshujing@163.com (S.R.)

**Keywords:** Pd@Pt core-shell icosahedron, one-pot synthesis, oxygen reduction reaction

## Abstract

Enhancing the limited utilization and overall yield of Pt-based catalysts is essential for advancing proton exchange membrane fuel cell technology. Herein, we report a facile one-pot method that utilizes TEG as both a solvent and a reductant to efficiently synthesize a Pd@Pt core-shell icosahedron. By controlling the surface energy between Pd and Pt precursors, we achieved the formation of Pd@Pt core-shell icosahedra, resulting in a fourfold reduction in reaction time and an eightfold increase in yield. Moreover, the core-shell structures exhibited a significant enhancement in electrocatalytic activity, stability, and Pt utilization efficiency. In comparison to commercial Pt/C, the Pd@Pt core-shell icosahedron exhibited efficient mass activity (MA, 1.54 A mg^−1^_Pt_) and specific activity (SA, 2.24 mA cm^−2^_Pt_) at 0.9 V (vs. RHE), while demonstrating excellent stability with minimal loss of activity even after 10,000 potential cycles. The Pd@Pt icosahedra configuration integrates the advantages of multiply twinned nanostructures, leading to rich electrochemical active surface sites and fast charge transport, thereby improving its catalytic performance and long-term stability during electrocatalytic reactions.

## 1. Introduction

Proton exchange membrane fuel cells (PEMFCs) face a significant challenge, primarily due to the requirement to incorporate substantial amounts of Pt-based catalysts into the cathode in order to accelerate the slow kinetics of the oxygen reduction reaction (ORR) [1,2,3,4,5]. However, the limited availability of precious metals such as Pt, along with rising costs and sustainability concerns, presents a substantial barrier to the large-scale commercialization of PEMECs technology. It is a significant challenge to reduce the amount of Pt while maintaining the catalyst’s performance. An effective approach to addressing this challenge involves improving the Pt utilization efficiency through the reduction of Pt-based catalysts’ particle size and morphology, which in turn improves the dispersion of Pt atoms [6,7,8,9,10]. Consequently, commercial ORR catalysts typically consist of Pt particles with dimensions in the range of 2 to 5 nm. Although these small Pt particles are extensively utilized, optimizing their specific activity remains a significant challenge [11,12,13,14].

Recent years have seen the investigation of several alternative strategies aimed at enhancing Pt utilization efficiency, by simultaneously improving the structure of Pt-based catalysts and increasing their specific activity [15,16,17,18,19,20,21,22,23]. One such strategy involves surface structure engineering through facile-controlled synthesis, aimed at optimizing Pt utilization and minimizing the quantity of Pt required. Alloy catalysts have garnered widespread attention from researchers due to their excellent ORR performance, such as PtAg/3DMGS [24], a Pt-Ni octahedron [25], and Pd-Pt Alloy hollow nanostructure [26]. However, alloy-based catalysts may experience alloying effects that reduce the density of active sites or disrupt their electronic structure, resulting in decreased catalytic performance and stability. In contrast, the deposition of Pt atoms as a conformal shell onto nanoparticles composed of less costly and/or more abundant metals represents a key strategy [27,28,29,30,31,32,33]. To achieve this, both chemical and electrochemical methods were developed to form Pt conformal shells, each just a few atomic layers thick on Pd-based nanoparticles [34,35]. The resulting bimetallic nanoparticles exhibit enhanced specific activity toward the ORR. Despite these promising advancements, the conventional strategy for constructing such core-shell structures relies on a hard template approach and epitaxial growth strategy. This synthesis method is not only complex in terms of procedure but also inefficient in yield. Consequently, the production of high-quality nanocrystals as catalysts while maintaining rapid synthesis and enhancing yield remains a considerable challenge [36].

Herein, we have innovatively developed a one-pot method that utilizes TEG as both a solvent and a reductant to rapidly produce a Pd@Pt core-shell icosahedron. Interestingly, controlling the surface energy between Pd and Pt precursors to form a Pd@Pt core-shell icosahedron resulted in a fourfold reduction in reaction time and an eightfold increase in yield, along with a substantially enhanced electrocatalytic performance. The Pd@Pt core-shell icosahedron showed the highest mass activity (MA) of 1.54 A mg^−1^_Pt_ and a specific activity (SA) of 2.24 mA cm^−2^_Pt_ at 0.9 V versus the reversible hydrogen electrode (vs. RHE), which is 6.2 and 7.0 times higher than that of commercial Pt/C (0.24 A mg^−1^_Pt_ and 0.32 mA cm^−2^_Pt_, respectively). Impressively, the Pd@Pt exhibited superior electrocatalyst stability, with only a 2.6 mV shift in ORR polarization curves (maintaining 90.3% MA) over 10,000 potential cycles.

## 2. Materials and Methods

### 2.1. Materials

Platinum (II) acetylacetonate (Pt(acac)_2_, Aladdin, Shanghai, China, 97%), perchloric acid (HClO_4_, Aladdin, Shanghai, China, 70.0~72.0%), potassium hydroxide (KOH, Aladdin, Shanghai, China, ≥85%), Ethanol (C_2_H_6_O, Aladdin, Shanghai, China, ≥99.7%), aqueous hydrazine solution (N_2_H_4_, Aladdin, Shanghai, China, 35 wt %), methanol (CH_4_O, Aladdin, Shanghai, China, 99.5%), triethylene glycol (TEG, Aladdin, Shanghai, China, ≥99.0%), acetone (C_3_H_6_O, Aladdin, Shanghai, China, ≥99.5%), Polyvinylpyrrolidone (PVP, Aladdin, Shanghai, China, Mw = 58,000), Sodium tetrachloropalladate (II) (Na_2_PdCl_4_, Macklin, Shanghai, China, 98%), Nafion (Macklin, Shanghai, China, 5 wt%), Commercial Pt/C catalyst (Sinero technology Co., Ltd., Suzhou, China, 20 wt%). Deionized water (Ulupure, Chengdu, China, 18.2 MΩ cm^−1^) was utilized in all experiments.

### 2.2. Methods

#### Preparation of Pd@Pt Core-Shell Icosahedron

Typically, 50 mg of PVP were dissolved in 7 mL of TEG within a glass vial, and the resulting solution was then heated to 160 °C in an oil bath, while magnetic stirring was continued for 10 min. In a separate glass vial, 36 mg of Na_2_PdCl_4_ and 12 mg of Pt(acac)_2_ were combined with 3 mL of TEG. This mixture was stirred for 30 min to ensure thorough dissolution of the metal precursors. Once the metal precursor solution was prepared, it was rapidly added to the vial containing the PVP-TEG solution. The combined solution was then subjected to a reaction at 160 °C for 90 min, with continuous stirring under the same conditions. After this reaction time, the process was terminated by quenching the mixture in an ice-water bath to halt any further chemical reaction. The product formed during the reaction was isolated through centrifugation, followed by washing once with acetone and twice with ethanol to remove any remaining impurities to obtain the Pd@Pt nanostructures.

### 2.3. Characterization

X-ray diffraction (XRD) characterization was performed on a D8 X-ray diffractometer (Bruker, Karlsruhe, Germany) with Cu-Kα radiation (λ = 1.54056 Å) operated at 40 kV and 40 mA. Transmission electron microscopy (TEM) was generated utilizing a JEOL JEM-1400 Plus microscope (Beijing, China). High-resolution transmission electron microscopy (HRTEM) were obtained on a Talos F200S-type field emission transmission electron microscope (Hillsboro, OR, USA). The actual Pt loading was quantified using inductively coupled plasma-optical emission spectrometry (ICP-OES) on a prodigy instrument (Hubson, NH, USA).

### 2.4. Electrochemical Measurements

All electrochemical measurements were conducted using a CS310H electrochemical workstation equipped with a three-electrode system and a rotating disk electrode (RDE) system. A graphite rod served as the counter-electrode, while Ag/AgCl was used as the reference electrodes. The glassy carbon electrode (Ø = 5 mm, S = 0.196 cm^−2^) functioned as the working electrode. The glassy carbon electrode was first polished by alumina powder with diameters of 1.5, 0.5, and 0.05 µm for 15 min and subsequently rinsed by sonicating in isopropyl and deionized water. Then, 0.98 mg of carbon-loaded catalyst was dispersed in 20 µL of Nafion ionomer (5wt%, Macklin) and 980 µL of ethanol solution (AR, ≥99.7%) to form the catalyst ink, which was then applied onto the surface of the glassy carbon electrode by dispensing 5 µL of the ink with a micropipette after 30 min of sonication in an ice bath. The catalyst ink was dried to form a high-quality catalyst thin film for electrochemical measurements, which were conducted at a rotation rate of 20 rpm for no less than 15 min at room temperature. The electrochemical tests for the Pd@Pt and Pt/C (20 wt%, JM) catalysts were measured under the same experimental conditions. The Pt loading for all the catalysts was maintained at 6.5 µg cm^−2^, with all loading masses normalized relative to the geometric electrode area of 0.196 cm^−2^.

The catalysts on the working electrodes were subjected to sonication and subsequently dispersed in ethanol to examine the morphological changes after the durability tests. Cyclic voltammograms (CVs) were recorded by scanning between 0.03 and 1.1 V versus a reversible hydrogen electrode (RHE) at a scan rate of 50 mV s^−1^ in N_2_-saturated 0.1 M HClO_4_ electrolyte solution. Linear scanning voltammetry (LSV) was performed from 0.2 to 1.1 V versus RHE at a sweep rate of 10 mV s^−1^ in O_2_-saturated 0.1 M HClO_4_ electrolyte solution with a rotation rate of 1600 rpm. Electrochemical impedance spectroscopy (EIS) was performed on various samples across the frequency range of 100 kHz to 0.1 Hz, employing an alternating voltage with an amplitude of 10 mV. ORR accelerated durability tests (ADTs) were performed in an aqueous solution of O_2_-saturated 0.1 M HClO_4_ for potential cycles at a sweep rate of 50 mV s^−1^.

The potential recorded by the Ag/AgCl electrode could be converted to the RHE potential using the following equation [37]:

E (vs. RHE) = E (vs. Ag/AgCl) + 0.197 + 0.0592PH(1)

The specific electrochemical active surface area (ECSA) was calculated using the following equation [38,39]:

ECSA = Q_H_/m × C (2)

where Q_H_ represents the charge associated with the adsorption of H_upd_, m is the Pt loading on the working electrode, and C (210 µC cm^−2^) is the charge corresponding to the monolayer adsorption of hydrogen on the Pt surface.

## 3. Results

**Figure 1:**
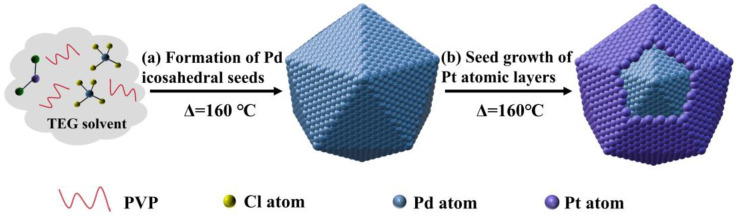
Schematic illustration of the growth process of the Pd@Pt icosahedron.

Figure 1 schematically illustrates the synthetic strategy used to fabricate the Pd@Pt core-shell icosahedron enclosed. Specifically, we demonstrated the configuration of the Pd@Pt core-shell icosahedron by the solvothermal reduction of Na_2_PdCl_4_ and Pt(acac)_2_ in a mixture of TEG and PVP at 160 °C. In this system, TEG served dual roles, acting both as the solvent and the reducing agent, and PVP acted as a stabilizing surfactant and co-reducing agent, ensuring proper control over the particle dispersion uniformity. We speculate that PdCl_4_^2−^ was reduced at a much faster rate than Pt(acac)_2_ under the same conditions. Due to the lower surface energy of Pd compared to Pt, PdCl_4_^2−^ was preferentially reduced in the presence of TEG (R_n_OH + M^2+^ + H_2_O → R_n-1_COOH + M^0^ +2H^+^), resulting in the aggregation of larger clusters, which later crystallized into a highly uniform and well-defined Pd icosahedron. Following this, Pt^2+^ in Pt(acac)_2_ was reduced to Pt atoms, which were then uniformly deposited onto the surfaces of the Pd icosahedron, leading to the formation of a homogeneous Pt shell. The notable difference in surface energy between Pd and Pt effectively impeded the migration of Pt atoms into the Pd core, thereby ensuring the establishment of a distinct core-shell structure [40,41].

**Figure 2:**
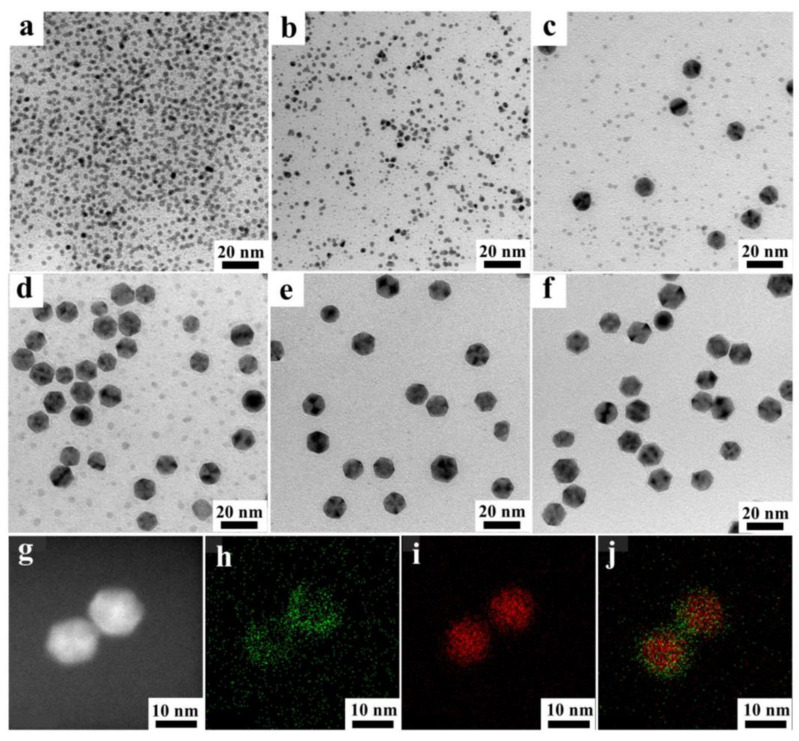
TEM images of Pd@Pt core-shell icosahedron with different reaction times at 90 °C: (**a**) 0.5 min, (**b**) 1 min, (**c**) 2 min, (**d**) 5 min, (**e**) 60 min and (**f**) 90 min. (**g**) STEM-EDS images and (**h**–**j**) EDS elemental mapping images of Pd@Pt core-shell icosahedron.

To better understand the structural evolution of a one-pot synthesis of a Pd@Pt icosahedron, the morphology and composition of the intermediate Pd@Pt icosahedron was extracted from the solution at various reaction times and analyzed using transmission electron microscopy (TEM) and scanning transmission electron microscopy-energy dispersive X-ray spectroscopy (STEM-EDS) mapping. The TEM images showed different stages of the Pd^2+^-Pd and Pt^2+^-Pt reduction between Na_2_PdCl_4_ and Pt(acac)_2_ (Figure 2). The solution immediately turned black when mixing Na_2_PdCl_4_ and Pt(acac)_2_ at 160 °C, implying a fast increase in the generation of nanocrystals. As shown in Figure 2a, nanoparticles formed at t = 30 s. After an additional 1 min, the nanoparticles transformed into an icosahedral shape, which subsequently agglomerated into larger clusters and eventually crystallized into well-defined icosahedral structures under the influence of TEG (Figure 2b). As the reaction time was prolonged to 2 min, the icosahedral shape tended to stay and grow (Figure 2c). In 5 min, the sample size increased to 12 nm, with only a few Pt depositions observed, indicating that Pd^2+^ primarily participated in the reduction process during this period (Figure 2d and Figure S1). As the reaction progressed, no notable changes in the sample size were observed (Figure 2e). At t = 90 min, the Pt deposition reaction was completed. When the reaction proceeded to 90 min, Pt atoms were uniformly deposited on the surface of Pd, leading to the formation of a high-quality Pd@Pt core-shell icosahedron (Figure 2f–h). It can be concluded that the Pd@Pt icosahedral structures were formed through rapid nucleation of Pd atoms during the reduction and deposition growth of Pt atoms (Figure 1). Compared to previous reports, the yield of the one-pot method increased nearly eight times and the reaction time was reduced almost fourfold [34].

From the perspective of reaction kinetics, the effects of temperature and PVP concentration on the Pd@Pt core-shell icosahedral structures were investigated. The experimental results at various temperatures are presented in Figure S2. At a reaction temperature of 100 °C, the product exhibited a small particle size of only 2 nm (Figure S2a), which can be attributed to the insufficient driving force or energy to effectively promote the formation of the icosahedral structure. As the reaction temperature increased to 160 °C, a more uniform icosahedral structure was observed (Figure S2b). As the temperature increased to 220 °C, additional morphologies such as tetrahedra and octahedra emerged at the edges of the icosahedral structure (Figure S2c), maybe resulting from excessively rapid reaction kinetics [42]. Similarly, the PVP concentration significantly influenced the morphology of the icosahedra. At a low PVP concentration (10 mg), insufficient surface stability resulted in the aggregation of the sample (Figure S3a). When the PVP concentration was increased to 50 mg, the dispersibility of the product improved significantly, leading to the formation of regular tetrahedral structures (Figure S3b). However, when the PVP concentration was further increased to 100 mg, small particles appeared around the samples (Figure S3c), likely resulting from the weak reducing properties of PVP’s hydroxyl groups, which promoted the aggregation of undeposited Pt atoms and the formation of Pt particles [43]. Therefore, precise control of the temperature and PVP concentration is crucial for the preparation of a high-purity Pd@Pt core-shell icosahedron.

**Figure 3:**
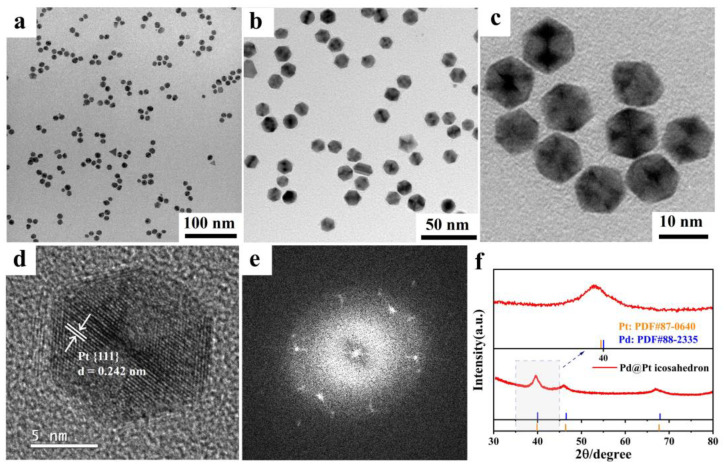
TEM images of Pd@Pt core-shell icosahedron with different magnifications. (**a**) 100 nm, (**b**) 50 nm, (**c**) 10 nm. HRTEM images of (**d**) Pd@Pt core-shell icosahedron. (**e**) FFT pattern of the image shown in (**d**). (**f**) XRD patterns of Pd@Pt core-shell icosahedron.

We further conducted analysis of the samples using scanning electron microscopy (SEM), TEM at various magnifications, and high-resolution transmission electron microscopy (HRTEM). TEM images clearly indicate that the majority of the samples exhibit a well-defined hexagonal morphology, typical of the Pd@Pt core-shell icosahedron. Importantly, the Pd@Pt core-shell structures maintained excellent dispersion throughout the sample, as evidenced by the uniform distribution of particles without noticeable agglomeration (Figure 3a–c and Figure S4). The size of the icosahedral nanoparticles was measured to be 11.5 ± 0.5 nm (Figure S5), which indicates that the synthetic approach employed effectively controlled both the particle size and distribution, contributing to the stability and even dispersion of the nanostructures.

HRTEM micrographs showed the characteristic {111} planes within the tetrahedral crystal domains, and the lattice constant was determined to be 0.242 nm (Figure 3d). The TEM image (Figure S6) clearly shows the fivefold axis of symmetry of the Pd@Pt core-shell icosahedron [44,45,46,47]. The fast Fourier transform (FFT) image provided further evidence supporting the presence of the twin crystal structure (Figure 3e). The phase compositions of the Pd@Pt core-shell icosahedron was further explored by the powder X-ray diffraction (XRD) patterns. As shown in Figure 3f, no distinct XRD peaks for Pt were observed, likely due to their overlap with Pd peaks [48]. Notably, three prominent peaks of the Pd@Pt icosahedron were observed at 66.9°, 46.1°, and 39.6°. Compared to the (220), (200), and (111) planes of Pd face-centered cubic (fcc) (JCPDS 88-2335), these peaks exhibit slight negative shifts, indicating that the Pd@Pt core-shell icosahedron may have undergone tensile strain effects. These observations further underscore the successful formation of the Pd@Pt core-shell icosahedral structure, which possesses distinct crystallographic characteristics that may influence its catalytic properties.

**Figure 4:**
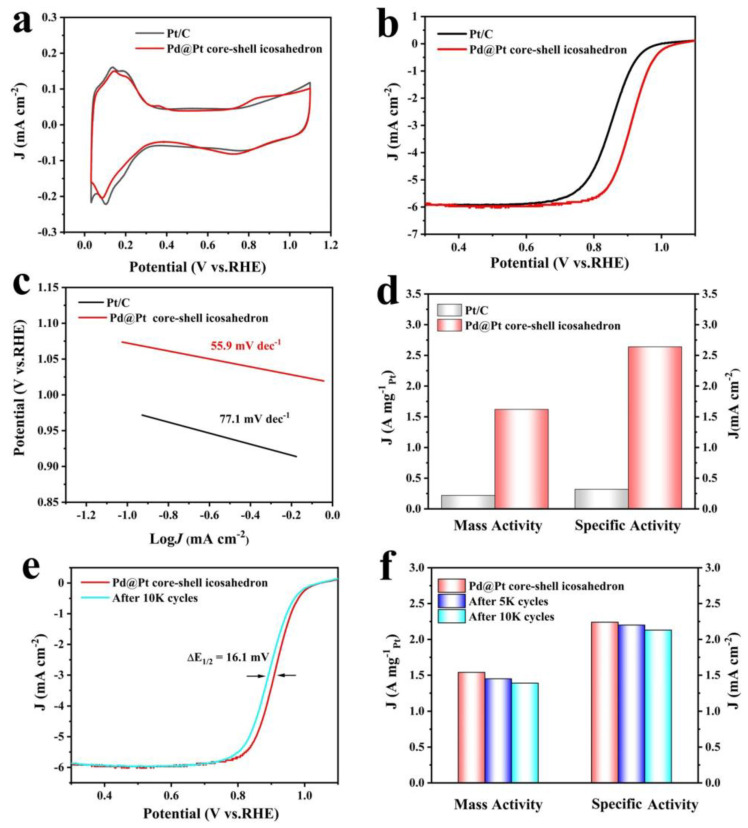
Electrocatalytic performance of Pd@Pt core-shell icosahedron and commercial Pt/C catalyst for ORR. (**a**) CV curves. (**b**) ORR polarization curves. (**c**) Tafel plots. (**d**) MA and SA. (**e**) ORR polarization curve evolutions for the Pd@Pt core-shell icosahedron before and after 10,000 potential cycles. (**f**) MA and SA for the Pd@Pt core-shell icosahedron before and after 10,000 potential cycles.

We then deposited the Pd@Pt core-shell icosahedron sample onto carbon to prepare the Pd@Pt core-shell icosahedron electrocatalysis and evaluated its ORR performance using the rotating disk electrode (RDE) method. The electrocatalytic performance of the Pd@Pt core-shell icosahedron was evaluated and compared with that of commercial Pt/C. Figure 4a illustrates the cyclic voltammetry (CV) curves of the samples, which were recorded in a N_2_-saturated 0.1 M HClO_4_ solution. The measurements were performed at a scan rate of 50 mV s^−1^ over a potential range of 0.2–1.1 V versus the RHE. The electrochemical active surface area (ECSA) of the Pd@Pt core-shell icosahedron was calculated to be 65.2 m^2^g^−1^_Pt_, which was considerably smaller than that of the commercial Pt/C, with an ECSA of 74.7 m^2^g^−1^_Pt_. Figure 4b presents the ORR polarization curves of the samples, recorded in an O_2_-saturated 0.1 M HClO_4_ aqueous solution using a glass carbon RDE at room temperature. The half-wave potential (E_1/2_) of the Pd@Pt core-shell icosahedron (E_1/2_ = 0.908 V) shows a significant positive shift compared with commercial Pt/C (E_1/2_ = 0.852 V), demonstrating the enhanced ORR catalyst activities of the Pd@Pt core-shell icosahedron. As shown in Figure 4c, the Tafel plots for specific activity display slopes of 55.9, 77.1 mV dec^−1^ for the Pd@Pt core-shell icosahedron and Pt/C electrocatalysts, respectively. The notably smaller Tafel slope observed for the Pd@Pt core-shell icosahedron indicates a significant enhancement in ORR kinetics [49,50]. The kinetic currents derived from the ORR polarization curves were determined using the Koutecky-Levich equation and subsequently normalized with respect to the ECSA and Pt mass to determine the SA and MA, respectively. At 0.9 V, the SA of the Pd@Pt core-shell icosahedron was 1.54 mA cm^−2^_Pt_, approximately 7.3 times higher than that of the Pt/C (0.21 mA cm^−2^_Pt_). As a pivotal indicator of the commercialization potential of a Pt-based catalysts, it is worth noting that the MA (1.86 A mg^−1^_Pt_) of the Pd@Pt core-shell icosahedron was 6.2 times higher than that of the Pt/C catalyst (0.30 A mg^−1^_Pt_) at 0.9 V versus RHE (Figure 4d). Electrochemical impedance spectroscopy (EIS) measurements reveal that the charge transfer resistance of Pd@Pt is higher than that of Pt/C (Figure S7). Consequently, we can infer that Pd@Pt demonstrates superior electron transfer efficiency compared to Pt/C [51]. The ORR activity of the Pd@Pt core-shell icosahedron was compared to the performance of Pd-Pt catalysts reported in recent years (Table S1); this reveals that the Pd@Pt core-−shell catalyst is among the highest-performing Pd-Pt catalysts.

The electrocatalytic durability of all the catalysts was evaluated by performing linear potential sweeps between 0.2 and 1.1 V versus RHE at a scan rate of 50 mV/s in O_2_-saturated 0.1 M HClO_4_ solutions. As shown in Figure 4e and Figure S8, the evolution of the CV and ORR polarization curves of the Pd@Pt core-shell icosahedron before and after potential cycling demonstrates that, after 10,000 potential cycles, the half-wave potential decreased by 16.1 mV when compared to the fresh sample. The SA and MA of the Pd@Pt core-shell icosahedron decreased by only 9.7% and 4.9% (Figure 4f), respectively. The TEM image revealed that the structural characteristics and sizes of the Pd@Pt core-shell icosahedron exhibited minimal changes after 10,000 cycles (Figure S9). In comparison, the commercial Pt/C exhibited a significantly larger negative shift (~71.2 mV) in the ORR polarization curves, accompanied by a 61.9% decrease in MA, a 46.4% reduction in SA, and pronounced carbon corrosion after 10,000 cycles (Figure S10).

## 4. Conclusions

In summary, we developed a general and robust approach for the one-pot method that leverages TEG as both a solvent and a reductant to efficiently synthesize a Pd@Pt core-shell icosahedron. By precisely controlling the surface energy between Pd and Pt precursors, we were able to facilitate the formation of these core-shell structures, leading to notable improvements, such as a fourfold reduction in reaction time and an eightfold increase in yield. The Pd@Pt core-shell icosahedra demonstrated significantly enhanced ORR activity and stability compared to state-of-the-art commercial Pt/C. Specifically, the as-prepared Pd@Pt core-shell icosahedra exhibited a MA of 1.54 A mg^−1^_Pt_ and a SA of 2.24 mA cm^−2^_Pt_ at 0.9 V versus RHE, which are approximately 6.2 and 7.0 times greater than those of commercial Pt/C, respectively. Impressively, the Pd@Pt core-shell icosahedron exhibits exceptional stability for ORR, with negligible activity decay and structural degradation over 10,000 cycles. This study demonstrates the engineering of core-shell bimetallic nanocrystals, utilizing surface energy differences between metals to enhance performance for ORR and other reactions. In addition, it provides valuable insights for the future development of electrocatalysts, with potential applications in renewable energy devices and beyond.

## Data Availability

The original contributions presented in this study are included in the article/Supplementary Materials. Further inquiries can be directed to the corresponding author.

## Supplementary Materials

The following supporting information can be downloaded at: https://www.mdpi.com/article/10.3390/ma18061279/s1, Figure S1. (a) HAADF-STEM images and (b–d) EDS elemental mapping images of the reaction to Pd@Pt core-shell icosahedron for 5 min; Figure S2. TEM images of Pd@Pt core-shell icosahedron with different reaction temperature. (a) 100 °C, (b) 160 °C, (c) 220 °C; Figure S3. TEM images of Pd@Pt core-shell icosahedron with different PVP content at 180 °C; (a) 10 mg, (b) 50 mg, (c) 100 mg; Figure S4. SEM images of Pd@Pt core-shell icosahedron; Figure S5. The size distribution histogram of Pd@Pt core-shell icosahedron; Figure S6. TEM image of an individual Pd@Pt core-shell icosahedron; Figure S7. EIS curves of Pt/C and Pd@Pt core-shell icosahedron; Figure S8. CV curve evolutions for Pd@Pt core-shell icosahedron before and after 10,000 potential cycles; Figure S9. TEM images of Pd@Pt core-shell icosahedron (a) before and (b) after 10,000 cycles’ durability test. TEM images showed negligible change in overall morphology or size of the Pd@Pt core-shell icosahedron after 10,000 cycles; Figure S10. The durability testing results for the Pt/C. (a) CV evolution before and after 10,000 potential cycles. (b) ORR polarization curve evolution before and after 10,000 potential cycles. (c) Mass activity and specific activity before and after different potential cycles. Table S1. Comparison of the ORR activity in our work with that of other catalysts reported in recent years. References [52,53,54,55,56,57,58,59,60,61,62] have been cited in Supplementary Materials.
