# Supplementary material for: One-Pot Synthesis of Pd@Pt Core-Shell Icosahedron for Efficient Oxygen Reduction
Source: Materials (Basel). 2025 Mar 13;18(6):1279. doi: 10.3390/ma18061279 (PMC11943620; doi:10.3390/ma18061279)
Supplement: Supplementary file 1 [file materials-18-01279-s001.zip › materials-3507902-supplementary.pdf]

Supporting Information

One-Pot Synthesis of Pd@Pt Core-Shell Icosahedron for Efficient Oxygen Reduction

Zisheng Tang, Dafu zhao, Xiaoqian Wang, Yanhui Jiao, Manrui Liu, Chengqi Liu, Qi Zhang, Shujing Ren, Yong Liu *

State Key Laboratory of Advanced Technology for Materials Synthesis and Processing, International School of Materials Science and Engineering (ISMSE), Wuhan University of Technology, Wuhan 430070, China; tangzs3076@163.com (Z.T.); dafu484@whut.edu.cn (D.Z); 303568@whut.edu.cn (X.W.); yanhui.jiao@outlook.com (Y.J.); liumanr14@163.com (M.L); liuchengqi42@163.com (C.L.); zq13307239180@163.com (Q.Z). rshujing@163.com (S.R);

* Correspondence: liuyong3873@whut.edu.cn

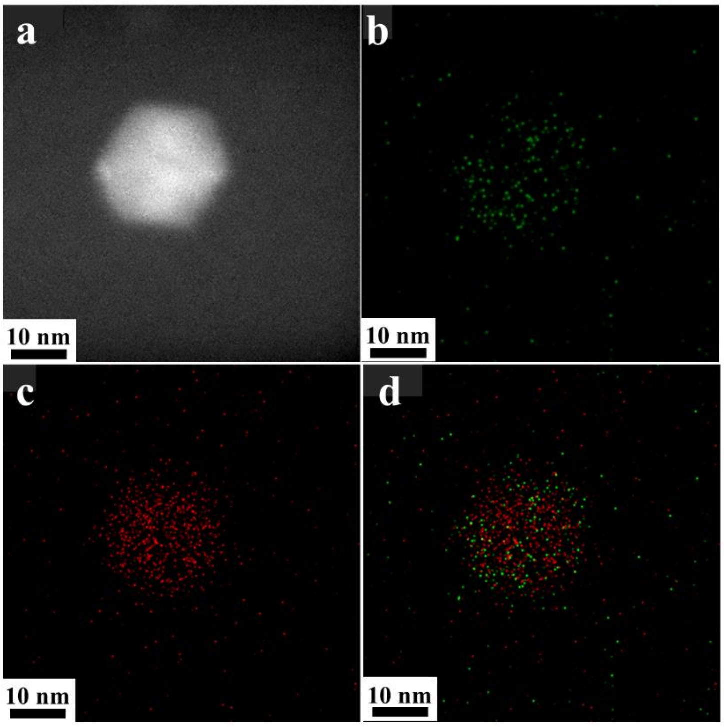

Figure S1. (**a**) HAADF-STEM images and (**b-d**) EDS elemental mapping images of the reaction to Pd@Pt core-shell icosahedron for 5 min.

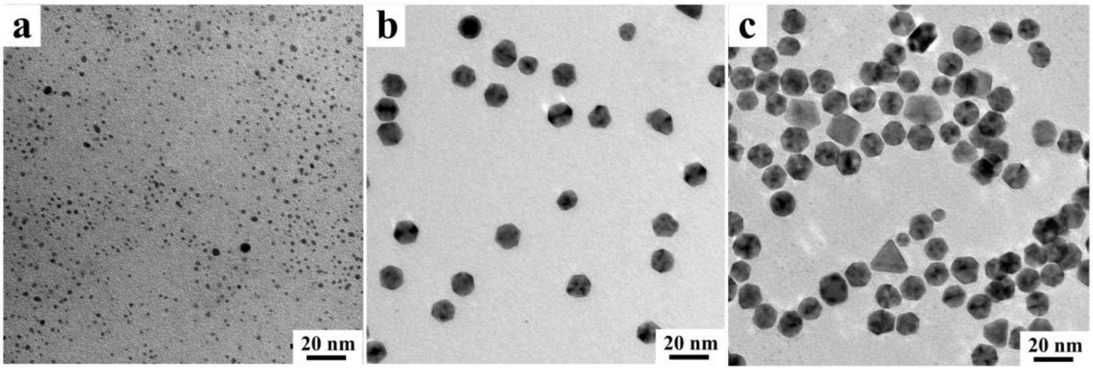

Figure S2. TEM images of Pd@Pt core-shell icosahedron with different reaction temperature. (**a**) 100 °C, (**b**) 160 °C, (**c**) 220 °C.

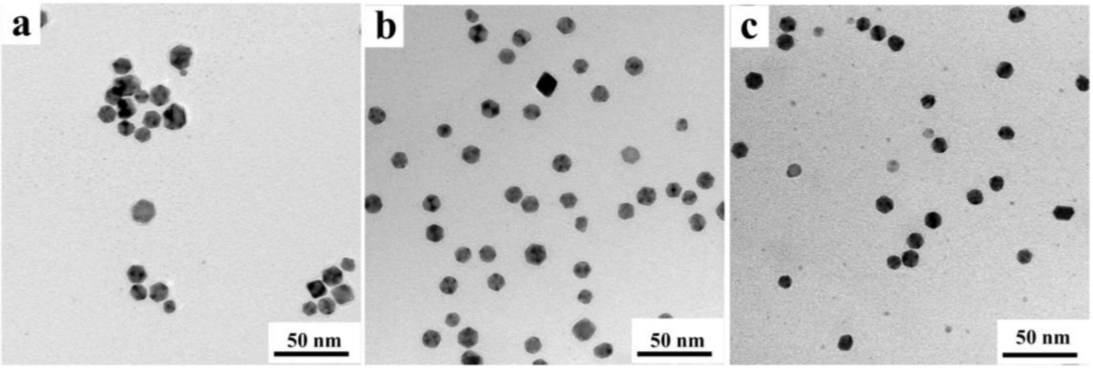

Figure S3. TEM images of Pd@Pt core-shell icosahedron with different PVP content at 160 °C. (**a**) 10 mg, (**b**) 50 mg, (**c**) 100 mg.

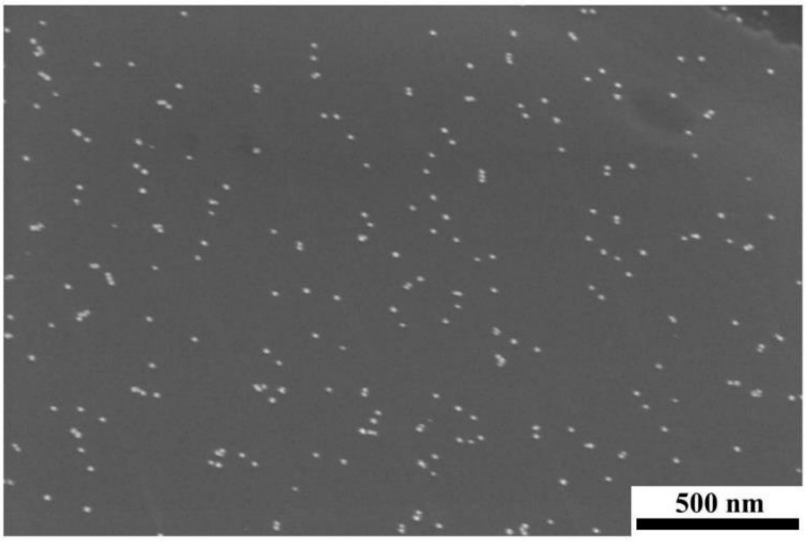

Figure S4. SEM images of Pd@Pt core-shell icosahedron.

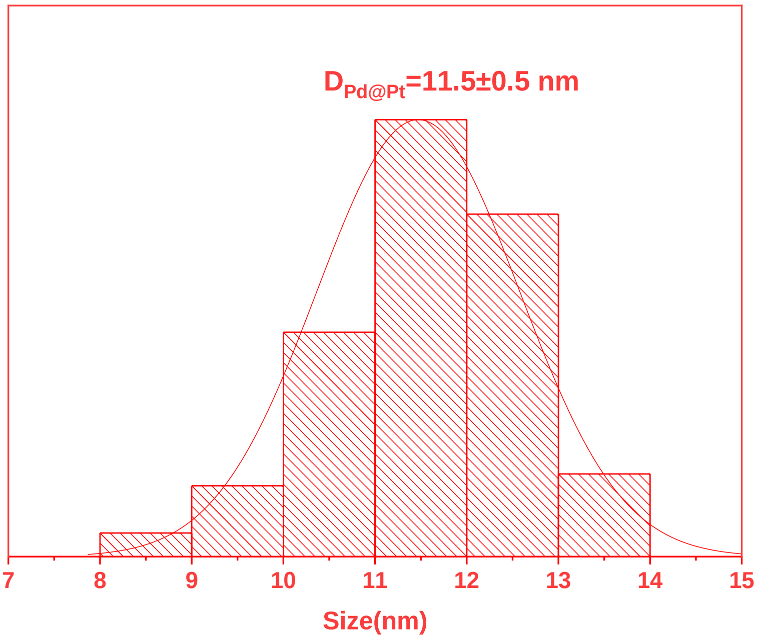

Figure S5. The size distribution histogram of Pd@Pt core-shell icosahedron.

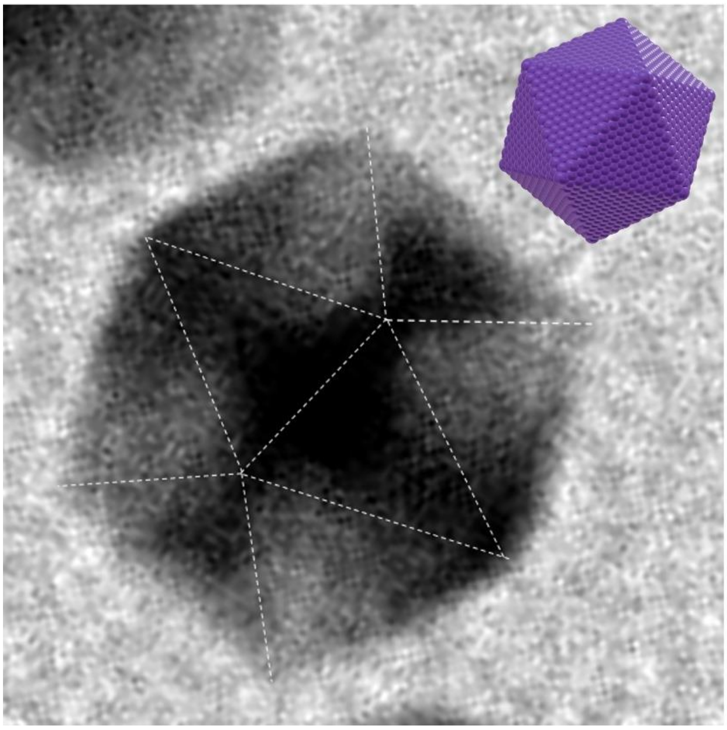

Figure S6. TEM image of an individual Pd@Pt core-shell icosahedron.

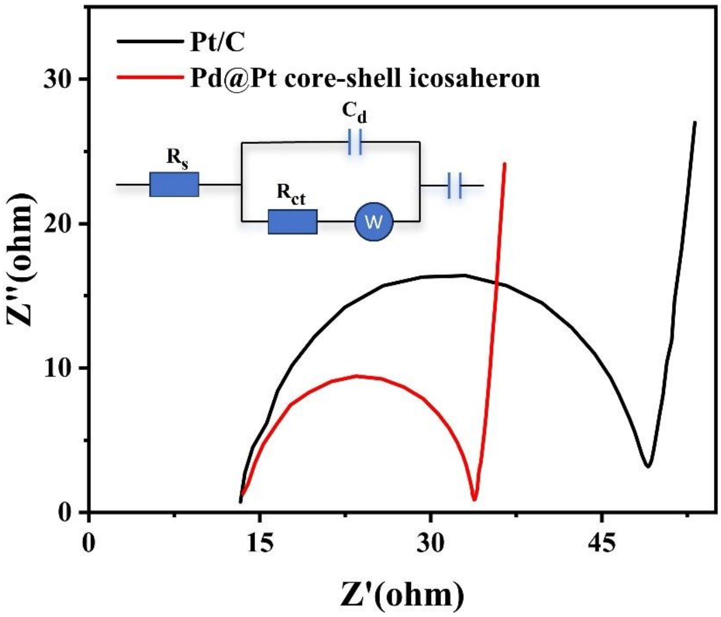

Figure S7. EIS curves of Pt/C and Pd@Pt core-shell icosahedron.

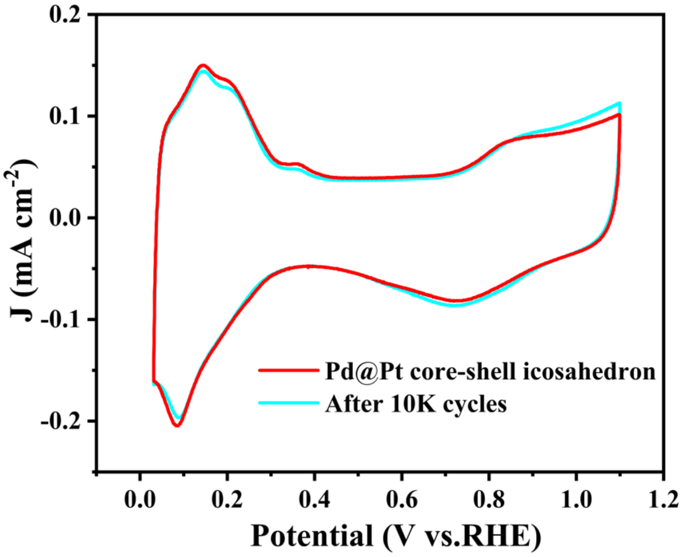

Figure S8. CV curve evolutions for Pd@Pt core-shell icosahedron before and after 10,000 potential cycles.

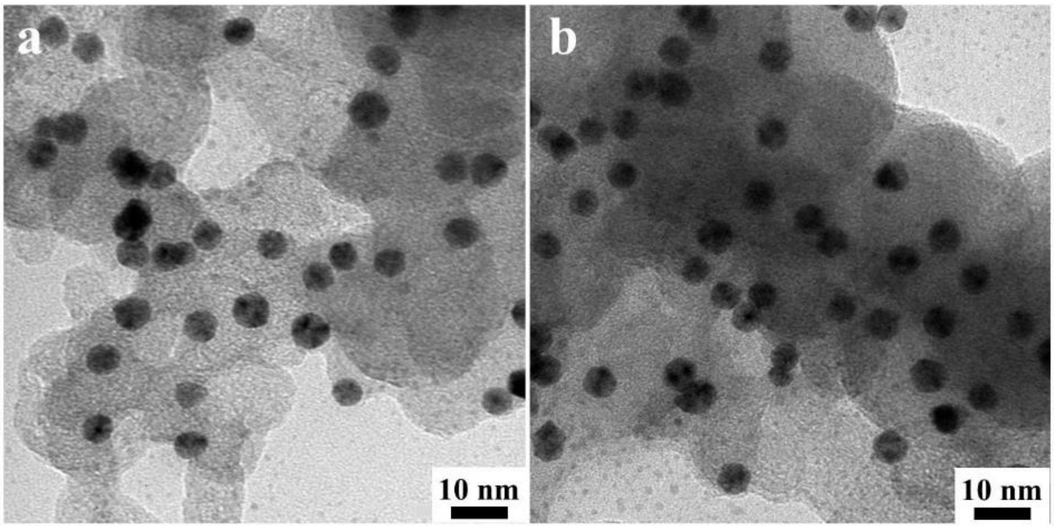

Figure S9. TEM images of Pd@Pt core-shell icosahedron (**a**) before and (**b**) after 10,000 cycles' durability test. TEM images showed negligible change in overall morphology or size of the Pd@Pt core-shell icosahedron after 10,000 cycles.

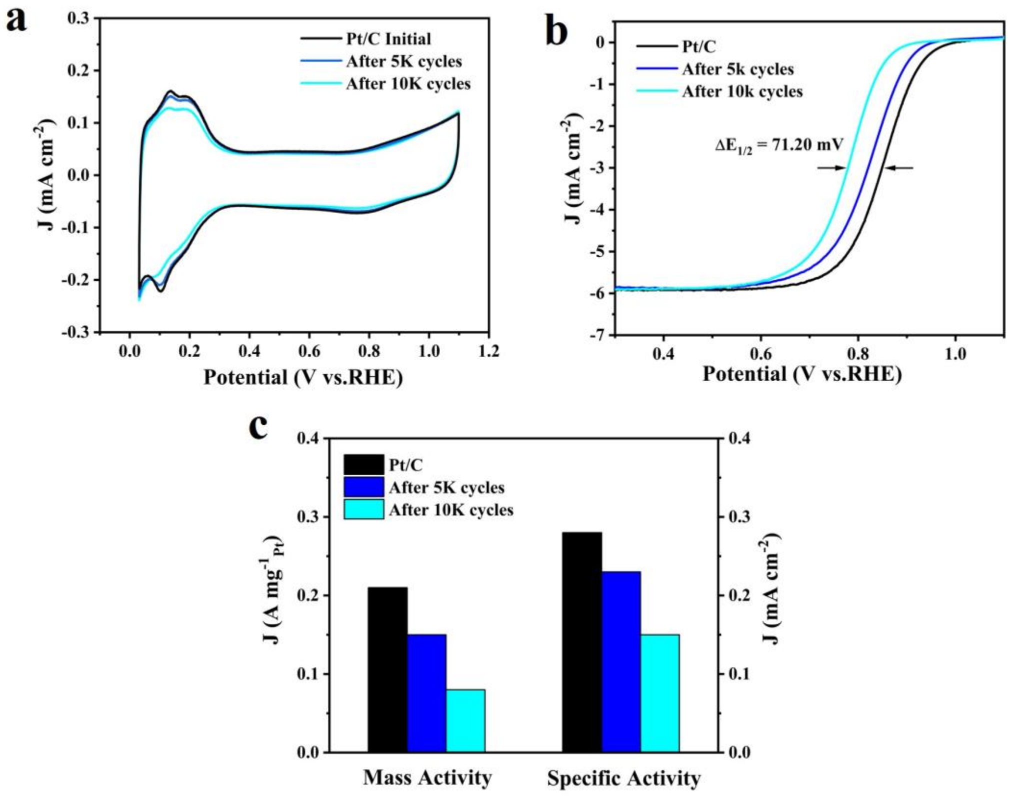

Figure S10. The durability testing results for the Pt/C. (a) CV evolution before and after 10,000 potential cycles. (b) ORR polarization curve evolution before and after 10,000 potential cycles. (c) Mass activity and specific activity before and after different potential cycles.

Table S1. Comparison of the ORR activity in our work with that of other catalysts reported in recent years.

Catalyst	ECSA ($m^2g^{-1}Pt$)	Mass activity ($A\ mg^{-1}_{Pt}$)	Specific activity ($mA\ cm^{-2}$)	Reference
**Pd@Pt icosahedron**	**65.2**	**1.54**	**2.24**	**This work**
Pd-Pt great icosahedra	124.1	1.23	0.99	[1]
Pd@Pt core-shell concave decahedra	95.9	1.60	1.66	[2]
Pt-Pd concave nanocubes	45.2	0.82	1.93	[3]
Pd@Pt core-shell concave nanocubes	61	0.84	1.38	[4]
Pd@Pt core-shell nanowires	202.5	0.81	0.4	[5]
Pd-Pt octahedra nanocages	40.3	0.76	1.9	[6]
Pd@Pt core-shell nanoparticles	48.82	0.58	0.44	[7]
Pt-Pd popcorns	47.4	0.57	0.52	[8]
Pt-Pd bimetallic nanocrystals	49.2	0.52	1.89	[9]
$Pd@Pt_{2\text{-}3L}$ octahedra	53.6	0.49	0.91	[10]
Pt-Pd nanodendrites	92.4	0.40	0.44	[11]
Pt@Pd core-shell icosahedra	38.8	0.29	0.74	[12]
Pd@Pt core-shell nanodots	84.38	0.20	0.24	[13]

References

1. Liu, M. K.; Lyu, Z. H.; Zhang, Y.; Chen, R. H.; Xie, M. H.; Xia, Y. N. Twin-Directed Deposition of Pt on Pd Icosahedral Nanocrystals for Catalysts with Enhanced Activity and Durability toward Oxygen Reduction. *Nano Lett.* **2021,** 21, 2248-2254.
2. Wang, X.; Vara, M.; Luo, M.; Huang, H. W.; Ruditskiy, A.; Park, J.; Bao, S. X.; Liu, J. Y.; Howe, J.; Chi, M. F.; Xie, Z. X.; Xia, Y. N. Pd@Pt Core-Shell Concave Decahedra: A Class of Catalysts for the Oxygen Reduction Reaction with Enhanced Activity and Durability. *J. Am. Chem. Soc.* **2015,** 137, 15036-15042.
3. Wu, R. F.; Tsiakaras, P.; Shen, P. K. Facile synthesis of bimetallic Pt-Pd symmetry-broken concave nanocubes and their enhanced activity toward oxygen reduction reaction. *Appl. Catal. B-Environ.* **2019,** 251, 49-56.
4. Lee, S. R.; Park, J.; Gilroy, K. D.; Yang, X.; Figueroa-Cosme, L.; Ding, Y.; Xia, Y. N. Palladium@Platinum Concave Nanocubes with Enhanced Catalytic Activity toward Oxygen Reduction. *ChemCatChem*. **2016,** 8, 3082-3088.
5. Li, H. H.; Ma, S. Y.; Fu, Q. Q.; Liu, X. J.; Wu, L.; Yu, S. H. Scalable Bromide-Triggered Synthesis of Pd@Pt Core-Shell Ultrathin Nanowires with Enhanced Electrocatalytic Performance toward Oxygen Reduction Reaction. *J. Am. Chem. Soc.* **2015,** 137, 7862-7868.
6. Hong, J. W.; Kang, S. W.; Choi, B. S.; Kim, D.; Lee, S. B.; Han, S. W. Controlled Synthesis of Pd-Pt Alloy Hollow Nanostructures with Enhanced Catalytic Activities for Oxygen Reduction. *ACS Nano*. **2012,** 6, 2410-2419.
7. Choi, R.; Choi, S. I.; Choi, C. H.; Nam, K. M.; Woo, S. I.; Park, J. T.; Han, S. W. Designed Synthesis of Well-Defined Pd@Pt Core-Shell Nanoparticles with Controlled Shell Thickness as Efficient Oxygen Reduction Electrocatalysts. *Chem.-Eur. J.* **2013,** 19, 8190-8198.
8. Ma, Y. X.; Yin, L. S.; Cao, G. J.; Huang, Q. L.; He, M. S.; Wei, W. X.; Zhao, H.; Zhang, D. G.; Wang, M. Y.; Yang, T. Pt-Pd Bimetal Popcorn Nanocrystals: Enhancing the Catalytic Performance by Combination Effect of Stable Multipetals Nanostructure and Highly Accessible Active Sites. *Small*. **2018,** 14, 10.
9. Cho, K. Y.; Yeom, Y. S.; Seo, H. Y.; Lee, A. S.; Do, X. H.; Hong, J. P.; Jeong, H. K.; Baek, K. Y.; Yoon, H. G. Fine-sized Pt nanoparticles dispersed on PdPt bimetallic nanocrystals with non-covalently functionalized graphene toward synergistic effects on the oxygen reduction reaction. *Electrochim. Acta*. **2017,** 257, 412-422.
10. Wang, X.; Park, J.; Zhang, L.; Xia, Y. N. Atomic layer-by-layer deposition of platinum on palladium octahedra for enhanced catalysts toward the oxygen reduction reaction. *Abstr. Pap. Am. Chem. Soc.* **2015,** 250, 1.
11. Xiong, X. L.; Chen, W. H.; Wang, W.; Li, J.; Chen, S. L. Pt-Pd nanodendrites as oxygen reduction catalyst in polymer-electrolyte-membrane fuel cell. *Int. J. Hydrog. Energy*. **2017,** 42, 25234-25243.
12. He, D. S.; He, D. P.; Wang, J.; Lin, Y.; Yin, P. Q.; Hong, X.; Wu, Y.; Li, Y. D. Ultrathin Icosahedral Pt-Enriched Nanocage with Excellent Oxygen Reduction Reaction Activity. *J. Am. Chem. Soc.* **2016,** 138, 1494-1497.
13. Li, S.; Liu, J. W.; Zhu, G. L.; Han, H. Y. Pd@Pt Core-Shell Nanodots Arrays for Efficient Electrocatalytic Oxygen Reduction. *Acs Applied Nano Materials*. **2019,** 2, 3695-3700.
